# Illustrations of Modern Memerism from Personal Investigation

**Published:** 1846-01

**Authors:** 


Art. V.—*Illustrations of Modern Mesmerism from Personal Investigation.* By John Forbes, m.d. f.r.s., Physician to Her Majesty's Household.—*London,* 1845. 18mo, pp. 112.

It will not be expected that we are going to give any particular account, much less to offer any opinion of the merits of this little book. The subject of it, however, has of late years excited so much interest among the members of the medical profession, that we think it right that they should be made aware of the general nature of its contents. These are the following:

"I. Alexis. (Two Exhibitions.)
"II. Adolphe. (Trials A, B, C, D.)
"III. Lady-Somnambulist. (Trials E, F, G.)
"IV. Fraulein von Gönnern.) (Trials H, I. Remarks.)
"V. George Goble. (First day—four experiments.)
(Second day—three experiments.)
(Third day—two experiments.)
"VI. Miss Martineau's J.
"1. Miss Martineau's statement.
"2. Dr. Forbes's letter to the editor of the Athenæum.
"3. Dr. Brown's letter to Dr. Forbes.
"4. Extract from Mr. Greenhow's statement.
"5. Attested statement of Barbara Cole."

The whole consists of a republication of papers that originally appeared in the 'Medical Gazette' and Athenæum. The subjoined extract from the preface will show the object of the publication.

"How far the anticipations of my friends are correct, as to the more general interest likely to be taken in these Papers,—remains to be seen; but, slight as they are, I am disposed to believe that they may be of some benefit not merely to the public, but to the Mesmerists themselves. If received simply as specimens or illustrations of the sort of things which mesmeric professors daily hold forth to the world, and which the world receives, as marvels of the highest order and as truths admitting of no question, they must surely give rise to reflections that may lead to some beneficial results.

"If the professors, on further consideration of the subject, do not condescend to supply the public with evidence of a more satisfactory kind, the public must cease to be satisfied with the kind of evidence they do supply.

"Every one who has paid attention to the proceedings of professed mesmerists, —even those of the highest class, the members of the medical profession,—must now be thoroughly convinced of the absolute necessity of their changing the plan they have hitherto pursued, if they expect to see mesmerism regarded as a branch of human knowledge deserving the attention of scientific men. So long as they refuse to adopt the rigid system of observation required in the sciences, and repudiate all the ordinary rules of induction and rational inference deemed essential to establish facts in other departments of knowledge, they have no right to quarrel with those who persist in disbelieving—or who, at least, refuse to admit as truths,—things, most marvellous in themselves, which, if true, are, to say the least, in nowise proved to be so, and which, for the most part, have no other evidence in their favour than the bare assertions of ignorant, interested, and, it may be, very unprincipled persons. No one conversant with these proceedings, as hitherto conducted, can deny that few, if any, of the greatest marvels recorded by the mesmerists, and promulgated as unquestionable facts, repose on more sound foundations than, before trial, seemed to support those which the investigations detailed in the following pages proved to be utterly baseless and false. As *all*, then, *may* be untrue, are we not authorised to demand a new course of inquiry, or a new series of evidences, before we are called upon to admit the truth of Clairvoyance and the other transcendental phenomena of mesmerism? Are we not justified, for the future, in refusing to receive from the mesmerists marvellous statements as truths and facts, unless it is, at the same time, proved to be *impossible* to explain or account for them, on other, ordinary, or what may be called natural principles?

"It is also hoped that the perusal of the exposures contained in this little book, may teach a useful lesson to those numerous unscientific persons, who are accustomed to attend mesmeric exhibitions, public or private, from motives of rational curiosity, or with the commendable object of investigating what seem important truths. Such persons, it is believed, must now feel convinced that no reliance whatever is to be placed on the results presented at such exhibitions, as evidencing the truth and powers of mesmerism. As these results are witnessed by the ordinary visitor, it is quite impossible to discriminate the true from the false. The coarsest jugglery may pass with the honest spectator, seated at a distance from the scene of action, for mysterious and awful truths. If Herr Döbler and Monsieur Phillippe can puzzle and perplex a whole theatre, surely George Goble may bamboozle the erudite captain and the six ladies on Mr. Vernon's back seats!" (Preface, pp. vi-ix.)

